# Perspective on the Use of Innovative Surveillance Strategies Implemented for COVID-19 to Prevent Mosquito-Borne Disease Emergence in French Polynesia

**DOI:** 10.3390/v14030460

**Published:** 2022-02-24

**Authors:** Maite Aubry, Van-Mai Cao-Lormeau

**Affiliations:** Laboratory of Research on Infectious Vector-Borne Diseases, Institut Louis Malardé, Papeete 98713, French Polynesia; mlormeau@ilm.pf

**Keywords:** COVID-19, SARS-CoV-2, arbovirus, French Polynesia, epidemiology, surveillance, travelers

## Abstract

In French Polynesia, following the emergence of the severe acute respiratory syndrome coronavirus 2 (SARS-CoV-2) in March 2020, several control measures were implemented to prevent virus spread, including a population lockdown and the interruption of international air traffic. SARS-CoV-2 local transmission rapidly stopped, and circulation of dengue virus serotypes 1 and 2, the only arboviruses being detected at that time, decreased. After the borders re-opened, a surveillance strategy consisting of the testing by SARS-CoV-2 RT-PCR of travelers entering French Polynesia, and isolating those with ongoing infection, was implemented. This strategy proved efficient to limit the introduction of SARS-CoV-2, and should be considered to prevent the importation of other pathogens, including mosquito-borne viruses, in geographically isolated areas such as French Polynesia.

## 1. Introduction

**Figure 1:**
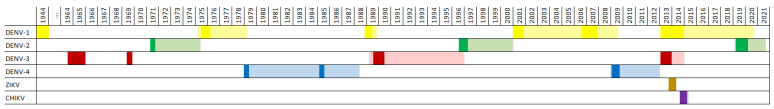
Circulation of the four dengue virus serotypes, Zika virus and chikungunya virus in French Polynesia, 1944–2021. DENV: dengue virus; ZIKV: Zika virus; CHIKV: chikungunya virus. Epidemic and inter-epidemic periods are indicated by bright and pale colors, respectively.

French Polynesia is a French overseas collectivity in the Southeast pacific, with ≈280,000 inhabitants distributed on 74 of the 119 islands. Like in other tropical areas, arthropod-borne viruses (arboviruses) are major public health threats in French Polynesia, particularly those transmitted by mosquitoes of the *Aedes* (*Ae.*) genus, *Ae. aegypti* and *Ae. polynesiensis* [1]. Since the 1940s, epidemics caused by the four serotypes of dengue virus (DENV-1 to -4), Zika virus (ZIKV), and chikungunya virus (CHIKV) have been recorded (Figure 1) [2,3]. The epidemiology of DENV was characterized by the circulation of a single serotype until 2013 when, for the first time, sustained co-circulation of different DENV serotypes (DENV-1 and DENV-3), and cases of ZIKV infection, were reported. The following year, the first cases of CHIKV infection were diagnosed. Since August 2020, DENV-2 has been the only arbovirus detected [4].

The first case of infection by the severe acute respiratory syndrome coronavirus 2 (SARS-CoV-2) was reported in French Polynesia in March 2020 [5]. As of 24 August 2021, a total of 40,178 cases of coronavirus disease 2019 (COVID-19) caused by SARS-CoV-2 had been recorded [6]. Here we present the control measures implemented in French Polynesia to fight against the spread of COVID-19 and their impact on the epidemiology of mosquito-borne diseases. We also discuss the possibility of adapting the innovative strategies implemented for COVID-19 surveillance to prevent future arbovirus outbreaks.

## 2. COVID-19 Control and Surveillance Measures

Subsequent to the emergence of COVID-19 in French Polynesia, a population lockdown was announced on 20 March, and lasted until 21 May 2020 [7]. Following this, to limit the transmission of SARS-CoV-2, people’s gatherings were strictly regulated [8], and a curfew was maintained until 20 June 2021 [9]. All patients with symptoms of COVID-19 had the opportunity to be tested for free, as well as people who had been in close contact with confirmed cases of SARS-CoV-2 infection [10,11].

In addition to the lockdown, international air traffic was interrupted between 28 March and 14 July 2020. Local circulation of SARS-CoV-2 stopped and only 62 cases of infection were detected during this period [12]. Borders were re-opened to Europe and the USA on 15 July 2020 [13]. To prevent new viral introductions, travelers entering French Polynesia had the obligation to present a negative result in a SARS-CoV-2 RT-PCR test performed within the 3 days before departure. In addition, an innovative strategy consisting of using self-collected and pooled oral and nasal samples for RT-PCR-based surveillance of SARS-CoV-2 in travelers was implemented [14,15]. However, it could not prevent the re-introduction of SARS-CoV-2 in French Polynesia, and an outbreak started in August 2020 [12].

As of 15 February 2021, self-collected samples from 273 travelers were positive for SARS-CoV-2. Subsequent viral spike (S) gene sequencing revealed that at least six travelers had been infected by the alpha variant of concern (VOC) before entering French Polynesia. All positive travelers were immediately isolated to prevent the spread of the virus, and people who had been in close contact with them were also tested. This strategy enabled the limitation of the transmission of SARS-CoV-2 VOCs, with only nine local contaminations recorded on 25 April 2021 [16].

On 3 February 2021, because of the emergence of VOCs and the resurgence in the number of COVID-19 cases in Metropolitan France, international air traffic was stopped a second time in French Polynesia [17], while the incidence rate of SARS-CoV-2 infections was decreasing [18]. When the borders reopened on 1 May 2021, the obligation for travelers to present a negative result for a SARS-CoV-2 RT-PCR test, dating less than 3 days before departure, was maintained to prevent the re-introduction of VOCs [19]. Moreover, new surveillance measures were implemented to screen travelers upon arrival [20]. All travelers aged 6 years and over were tested at the airport using the Panbio^TM^ COVID-19 Ag Rapid Test Device (Abbott, Chicago, IL, USA). To confirm the result of the antigenic rapid test, a second swab was collected from each traveler at the airport, and samples from up to 10 travelers were subsequently pooled before being tested by SARS-CoV-2 RT-PCR at the Institut Louis Malardé (ILM, Papeete, French Polynesia). Travelers who obtained a negative result for the antigenic rapid test were allowed to leave the airport provided they had been vaccinated, whereas those who were found positive using either screening test (antigenic rapid test or RT-PCR) were immediately isolated. In addition to the test on arrival, unvaccinated adult travelers were required to self-quarantine and to obtain negative results for both antigenic rapid test and pooled-samples-based RT-PCR test performed at ILM 4 days and 8 days after arrival, before being allowed to move freely. Unvaccinated minor travelers aged from 6 to 17 years old were provided self-sampling kits at the airport to collect both nasal and oral swabs, 4 days and 8 days after arrival, and were not required to self-quarantine. Self-collected samples from minors were pooled and tested by SARS-CoV-2 RT-PCR at ILM. As a result of those surveillance measures, 45 travelers were found positive between 1 May and 25 July 2021 [11]. Re-testing at ILM of positive samples using SARS-CoV-2 variant-specific amplification kits (VirSNIP SARS-CoV-2 Spike N501Y, L452R, P681R, V1176F, D253G, E484K, H66D/del69/70, and del69,70+484K+501Y kits, TIB MOLBIOL, Berlin, Germany), and subsequent S gene sequencing, revealed that most travelers had been infected by VOCs. From mid-July 2021, an upsurge in COVID-19 cases, mainly caused by the delta VOC, has been observed [11].

## 3. Impact of COVID-19 on Mosquito-Borne Disease Epidemiology

Although medical resources have been heavily mobilized for the diagnosis of COVID-19, the surveillance of arbovirus infections has been maintained in French Polynesia. Since July 2020, low DENV transmission has been observed, and DENV-2 has been the only arbovirus identified [4]. Nevertheless, the actual number of DENV infections might have been underestimated as some symptoms of dengue (including fever, body aches, and headache) are similar to those of COVID-19. Consequently, practitioners may have prescribed screening tests for SARS-CoV-2 instead of for DENV. Moreover, patients with mild symptoms of dengue may have chosen not to attend a health center for fear of contracting COVID-19.

Given the geographic isolation of French Polynesia, arboviruses causing epidemics are introduced by travelers coming from countries where these pathogens are circulating [2,3]. Successive border closures between 28 March and 15 July 2020, and between 3 February and 1 May 2021, prevented the introduction of new arboviruses. After the reopening of borders, flights between French Polynesia and other tropical countries did not resume, thus limiting the risk of introduction of other arboviruses.

The last DENV-2 outbreak started in April 2019 [3], and the number of dengue cases has dramatically decreased from March 2020 [21]. The lockdown implemented from 20 March to 21 May 2020 restricted people’s circulation and gatherings and, consequently, probably contributed to reducing the spread of mosquito-borne viruses.

## 4. Strategies to Prevent the Emergence of Mosquito-Borne and Other Viral Diseases

Since the introduction of arboviruses in French Polynesia is related to travelers, implementing surveillance measures on arrival could be an effective method to prevent the emergence of new pathogens. Similarly to the strategies used for SARS-CoV-2 surveillance, a biological sample could be collected from each traveler at the airport to detect ongoing viral infections. Blood is the most common biological sample used for the diagnosis of arboviruses. However, collecting venous blood is an invasive procedure that might be very poorly accepted by travelers. Instead, capillary blood is easy to collect at the fingertip and may be better tolerated by travelers, especially children. Moreover, studies having compared the use of venous and capillary blood samples for the diagnosis of DENV and ZIKV infections showed that the duration of virus detection was longer in capillary blood [22,23]. As an alternative to blood collection, saliva sampling is non-invasive and was shown to be suitable for the detection of arboviruses such as DENV, ZIKV, and CHIKV [24,25,26,27]. In addition to the surveillance of arboviruses, saliva could be used to detect respiratory viruses such as influenza virus [28], which regularly causes epidemics in French Polynesia [29]. Finally, urine could also be a good alternative for the detection of arboviruses, as previously demonstrated [30,31]. However, collecting urine from all travelers at the airport may be challenging for both logistics and sanitary reasons (limited number of restrooms). Moreover, in contrast to blood or saliva, the supervision of the self-collection of urine sample is not possible.

In order to increase testing capacity, biological samples from several travelers should be pooled before molecular diagnostics, as previously described for the surveillance of SARS-CoV-2 [14,15]. Adjustments to the pooling protocol (i.e., selection of the number and volume of individual samples to pool) might be required depending on the type of sample used (venous blood, capillary blood, saliva, or other). Real-time RT-PCR is the most widely used molecular diagnostic test for the detection of viral infections. However, the number of pathogens that can be simultaneously screened in one run of RT-PCR is limited. For a broader surveillance of the pathogens introduced into French Polynesia, new molecular diagnostic tools capable of detecting a large number of viruses simultaneously from a single sample, such as the Luminex [32] or next generation sequencing (NGS) technologies [33], could be used. Although more complicated and expensive to implement than a multiplex real-time RT-PCR, the Luminex and NGS technologies have the advantage of being less time-consuming as a larger number of pathogens can be tested at the same time, and are particularly relevant for the surveillance of new pathogens, including arboviruses and respiratory viruses [34,35,36].

In contrast to SARS-CoV-2 and other respiratory viruses that are transmitted directly from human to human, arboviruses are transmitted by vectors capable of moving from one location to another. For instance, mosquitoes of the *Ae. aegypti* species (which transmit several arboviruses such as DENV, ZIKV, and CHIKV), can fly within an area of more than 800 m in diameter [37].Thus, the strategy of isolating infected travelers, as previously used for SARS-CoV-2 control, cannot be used to prevent transmission of mosquito-borne viruses, unless the location chosen for the quarantine is free of vectors.

The better strategy to stop the spread of arboviruses is through vector control. On arrival in French Polynesia, travelers could be made aware of individual protection methods against mosquito bites (the use of repellents, mosquito nets, etc.). Moreover, for travelers testing positive on arrival with an arbovirus-related infection, the health authorities could provide them and their entourage with a kit to protect against mosquito bites, and remind them of the rules to follow to avoid virus transmission. Mosquito control (perifocal insecticide treatment, clearance of domestic mosquito-breeding sites) and investigation of potential secondary cases could also be performed in all locations where travelers tested positive had stayed since their arrival in French Polynesia. In addition to the measures implemented to prevent the introduction and spread of arboviruses, mosquito control strategies such as the insect incompatibility technique, using the maternally inherited endosymbiotic Wolbachia bacterium, could be considered in the islands of French Polynesia to eradicate the vectors. As evidence, experimental field trials conducted on an islet of Tetiaroa (Society Islands, French Polynesia), that consisted in repeated weekly releases of *Ae. polynesiensis* male mosquitoes modified with an alternate Wolbachia type, proved efficient to reduce the mosquito population density long-term [1].

## 5. Conclusions

Although respiratory and mosquito-borne viruses have different modes of transmission, they have the common feature of being imported into French Polynesia by travelers. Once introduced, the transmission of these viruses is difficult to control using only prevention measures (such as barrier gestures or self-protection against mosquito bites). Consequently, as implemented for COVID-19, the surveillance of infections among travelers at the gateways to French Polynesia (airport, port) should be considered to limit the introduction and spread of infectious diseases.

## Data Availability

No new data were created or analyzed in this study. Data sharing is not applicable to this article.
